# Serum and Brain Metabolomic Variations Reveal Perturbation of Sleep Deprivation on Rats and Ameliorate Effect of Total Ginsenoside Treatment

**DOI:** 10.1155/2017/5179271

**Published:** 2017-08-16

**Authors:** Xiao-jun Gou, Fang Cen, Zi-quan Fan, Ying Xu, Hong-yi Shen, Ming-mei Zhou

**Affiliations:** ^1^Center for Chinese Medicine Therapy and Systems Biology, Shanghai University of Traditional Chinese Medicine, Shanghai 201203, China; ^2^Central Laboratory, Baoshan District Hospital of Integrated Traditional Chinese and Western Medicine of Shanghai, Shanghai 201999, China; ^3^Department of Physiology, Shanghai University of Traditional Chinese Medicine, Shanghai 201203, China; ^4^Research Center for Health and Nutrition, Shanghai University of Traditional Chinese Medicine, Shanghai 201203, China

## Abstract

Sleep loss or sleep deprivation (SD) refers to shorter sleep than average baseline need, and SD has been a serious problem of modern societies which affects health and well-being. *Panax ginseng* is a well-known traditional Chinese medicine (TCM). Our previous study has demonstrated that total ginsenosides (GS), the extracts from *Panax ginseng*, could effectively improve cognition and behavior on SD rats. However, little is known about its metabolomic study. In this study, serum and brain metabolomic method based on gas chromatography coupled with mass spectrometry (GC/MS) was employed to evaluate the efficacy and study the mechanism of GS on a rat model of SD. With pattern recognition analysis of serum and brain tissue metabolite profile, a clear separation of the model group and control group was acquired for serum and brain tissue samples; the MGS (model + GS) group showed a tendency of recovering when compared to control group, which was consistent with behavioral and biochemical parameters. 39 and 40 potential biomarkers of brain tissues and serum samples, respectively, were identified and employed to explore the possible mechanism. Our work revealed that GS has significant protective effects on SD, and metabolomics is a useful tool for evaluating efficacy and elucidating mechanism in TCM.

## 1. Introduction

Sleep deprivation (SD) is the inability of an individual to meet the normal sleep means due to environment or their own reason, generally refers to sleep less than 4 h in 24 h, and leads to a series of changes including emotion, learning and memory, immune function, physiological, psychological, and even behavior [1]. Lack of sleep, long-term SD, or insufficient rest or sleep is very common in modern society. Nearly one-third of adult sleep time is less than 6 hours on average [2]. The factors responsible for this change may include an increase in ambient light, the introduction of light, longer working days/working hours, class night shifts, and manufacturing and services extended to 24 hours of operation, as well as television and internet [3]. SD can cause changes in mood, learning, and memory and damage to the body's multiple systems and organs, such as the central nervous system, cardiovascular system, digestive system, respiratory system, endocrine system, immune system, and reproductive system [4–9]. Inf1uence of SD has been revealed by many studies; the methods used in SD research include psychological assessment, brain imaging, cerebral electrophysiology, and gene expression based on genomic and transcriptomic studies [10–12]. However, the pathophysiologic mechanism of these damages has not yet been fully elucidated. At present, adjuvant drugs for SD play a role in the central nervous system, such as amphetamine, caffeine, and modafinil [13]. These medications are often associated with a number of side effects, including excessive sleepiness, thought disorders, nocturnal wandering, agitation, and balance problems, thereby limiting clinical use [14]. Ginseng roots (*Panax ginseng* C. A Meyer), one of the best known traditional Chinese medicine (TCM) herbs, have been reported to display adaptogenic effects in the endocrine, immune, cardiovascular, and central nervous systems [15]. As one of the major adaptogens, ginseng extract showed modulation effects on the blood system after paradoxical SD [16]. Total ginsenosides (GS), the most important active ingredient of *Panax ginseng*, have shown multiple activities in the nervous system in the previous studies, such as protection on memory deficit [17] and antidepressant effect [18]. These findings clearly indicate that GS possesses therapeutic effects on pathophysiological changes induced by SD. Little is known about the precise mechanisms by which GS protects against SD. Therefore, in order to find out the pathophysiological changes of SD and intervention mechanism of GS, a new approach for diagnosing and monitoring disease profession based on a metabolomic study was proposed.

Metabolomics, a relative new field of systems biology, aims to quantitatively measure as many small molecular metabolites as possible in a given biological system in order to acquire an overview of metabolic or disease status and global biochemical events associated with a cellular or biological system [19]. Nowadays, metabolomics is regarded as a potentially powerful application in many fields of medical research, such as novel potential disease biomarker discovery [20], efficacy evaluation of TCM prescriptions [21], and safety assessment of TCM herbs [22]. Mass spectrometry (MS) and nuclear magnetic resonance (NMR) spectroscopy are two major analytical platforms of metabolomics. MS coupled with advanced chromatographic separation apparatuses, such as gas chromatography (GC) or liquid chromatography (LC), has become a powerful metabolomic tool, with a wide dynamic range and reproducible quantitative performance, for the measurement of complex biological samples. GC/MS, a robust analytical platform for quantification with good sensitivity, resolution, and reliability, has commercial databases in structure identification of candidate biomarkers [23] and has become a popular and useful analytical tool in metabolomic studies [24]. In this paper, GC/MS-based metabolomic platform will be applied to investigate the perturbation of SD, and with the perturbed metabolomic profiles, further attempts were made to explore the possible therapeutic mechanism of GS on SD rats.

## 2. Materials and Methods

### 2.1. Chemicals and Drugs

N,O-Bis(trimethylsilyl) trifluoroacetamide (BSTFA) with 1% trimethylchlorosilane (TMCS) of analytical grade was from Regis (Morton Grove, IL, USA). Methanol and chloroform of analytical grade were purchased from CNW Technologies GmbH (Düsseldorf, Germany). Methoxylamine hydrochloride and anhydrous pyridine were purchased from Sigma-Aldrich (St. Louis, MO, USA). Commercial kit used for determining adrenocorticotropic hormone (ACTH) was obtained from Phoenix Pharmaceuticals (Burlingame, CA, USA). Commercial kit used for determining thyroid-stimulating hormone (TSH) was purchased from R&D Systems (Minneapolis, USA). Commercial kit used for determining corticosterone (CORT) was obtained from Cayman Chemical Co. Ltd (MI, USA). Commercial kits used for determining, catecholamine (CA), testosterone, and serotonin were purchased from Cusabio Biotech Co. Ltd, (Wuhan, China). Total ginsenosides (GS, purity ≥ 80%, UV method) extracted from the roots of *P. ginseng* were purchased from Nanjing Zelang Biomedical Technology Co. Ltd. (Nanjing, China).

### 2.2. Experimental Animals

The study was conducted in accordance with the Guidelines for Animal Experimentation of Shanghai University of TCM (Shanghai, China). Forty-eight-week-old male Sprague Dawley rats (200 ± 20 g) were purchased from the Shanghai SLAC Laboratory Animal Co. Ltd. (Shanghai, China). The rats were housed at 24 ± 1°C with 45 ± 15% relative humidity under 12/12 h light-dark cycle. All animals were provided with a certified standard rat chow and tap water ad libitum.

### 2.3. Animal Treatment and Sample Collection

After 1-week acclimation, forty rats were randomly divided into 5 groups (*n* = 8) as follows: normal animal group (N), normal group administrated with the GS group (NGS), wide platform group (WP), SD group (M), and SD with GS intervention group (MGS). The groups of N, WP, and M received normal saline solution, while the groups of NGS and MGS received GS orally at a daily dose of 100 mg/kg of body weight from day 1 to day 8. The rats in the M and MGS groups were sleep-deprived from day 4 for a 120 h period using the columns-in-water model (modified multiple platform) as described previously [25]. In addition, in order to test the effect of stress of the environment, wide platforms (16 cm in diameter) were used in rats of WP on which the rats could sleep without falling into the water and stay for 120 h. Rats in N and NGS were given no treatment. All rats were euthanized at the end of day 8, and the arterial blood and brain tissues were collected immediately. The blood samples were centrifuged at 3000 ×g and 4°C for 15 min, and the supernatant (serum) was collected and stored at −80°C. Hippocampal tissue was isolated from the brain tissues and snapped frozen in liquid nitrogen immediately and stored at −80°C.

### 2.4. Morris Water Maze (MWM) Test

Spatial learning and memory were measured by the MWM test. The Morris water maze (MWM) consisted of a circular pool (160 cm diameter, 45 cm in height), painted black, and filled with unclear water maintained at 25 ± 1°C. The pool was geographically divided into four quadrants of equal size, and the quadrants were labeled as north, east, south, and west in turn. There was a square platform (10 cm diameter, 25 cm tall) under (~1.5 cm) the water surface in the center of the northeast quadrant. Surrounding the tank was a black curtain to remove all spatial cues, and the room was lit with overhead lighting. On the experimental day 3, rats were placed in the water maze for a trial test. After 10 min acclimation to the room, each rat was placed into the tank in turn at one of four locations (north, south, east, or west) and allowed a maximum of 60 sec to find the platform. If the rat did not find the platform within the limited time, it was guided by hand to the platform location. Once on the platform, each rat remained there for an additional 20 sec. Each rat in the testing group completed its trial in turn, and a total of two learning trials were conducted per day. Two hours after the 2nd trial, the animals were given the test trial for MWM, and the latent time for each rat to reach the platform was recorded. Same procedures were performed on day 4 to day 8.

### 2.5. Biochemical Analysis

Hippocampal tissue was homogenized with ninefold of ice-cold saline, and subsequently, the mixture was centrifuged at 16,000*g* and 4°C for 15 min. The supernatants were collected for the determination of nitrogen oxide (NO) levels. The level of NO was measured using the NO kit based on the concentration of nitrites, which were reduced by specific nitrate reductase from nitrates. The absorbance of samples was detected at 550 nm. The results are expressed as micromoles of NO/g protein. Results were obtained using the PowerWave™ XS microplate spectrophotometer from BioTek (Winooski, USA).

Plasma concentrations of adrenocorticotropic hormone (ACTH), thyroid-stimulating hormone (TSH), catecholamine (CA), testosterone, corticosterone (CORT), and serotonin were determined by automatic biochemistry analyzer using the commercial ELISA kits according to the protocols provided by the manufacturer.

### 2.6. Sample Preparation and Analysis by GC/MS

Serum and hippocampal tissue samples were prepared following our previously published protocols with minor modifications [26]. Analysis was performed on an Agilent 6890 gas chromatography system coupled to an Agilent 5975B inert MSD system (Agilent Technologies Inc., CA). The derivatives were separated by DB-5 ms fused-silica capillary column (30 m × 0.25 mm × 0.25 *μ*m; Agilent J&W Scientific, Folsom, CA) with helium as a carrier gas at a constant flow rate of 1.0 ml/min. Each 1 *μ*l aliquot of derivatives was injected, and the solvent delay time was 5 min. For serum sample analysis, the initial oven temperature was held at 80°C for 2 min, ramped to 240°C at a rate of 5°C/min, to 290°C at a rate of 25°C/min, and finally held at 290°C for 10 min. For brain tissue analysis, the initial oven temperature was held at 80°C for 2 min, ramped to 240°C at a rate of 6°C/min, to 280°C at a rate of 30°C/min, and finally held at 280°C for 10 min. The temperatures of injector, transfer line, and electron impact ion source were set to 270°C, 260°C, and 230°C, respectively. Electron impact ionization (70 eV) at full scan mode (m/z 30–600) was used.

### 2.7. Data Analysis

Data from behavioral and biochemical test were performed using the Statistical Package for SPSS 19.0 (SPSS, Chicago) and expressed as means ± SD. Statistical significance among the groups was analyzed by one-way analysis of variance (ANOVA). The result of *P* < 0.05 was considered to be statistically significant.

GC/MS data were converted into AIA format (NetCDF) files by Agilent GC-MS 5975 Data Analysis software, and pretreatment was conducted as previously described [27]. The peak table (named matrix X) file was imported to a commercial software Simca-P 11.5 (Umetrics AB, Umeå, Sweden). Principle component analysis (PCA) was performed on the mean-centered and UV-scaled data to visualize general clustering, trends, and outliers among all samples on the score plot. These differential metabolites selected from the orthogonal partial least squares discriminant analysis (OPLS-DA) model with VIP value (VIP > 1) are validated at a univariate level with Wilcoxon–Mann–Whitney test with a critical *P* value usually set to 0.05.

### 2.8. Metabolite Identification

The AMDIS software was applied to deconvolute mass spectra from raw GC/MS data, and the purified mass spectra were automatically matched with an author-constructed standard library including retention time and mass spectra. The peaks which were not matched from a standard library were introduced to the NIST MS 2.0 software for automatically searching compound information from the NIST 11 library and Golm Metabolome Database. The searching results with match similarity larger than 80% will be accepted as candidate compounds. The Kovats retention indexes of remaining candidate compounds were calculated and compared with a NIST RI database and available internet database such as MassBank.

## 3. Results

### 3.1. Behavioral Study

The ability of rat spatial learning and memory tested by the Morris Water Maze (MWM) experiment was listed in Table 1. Before SD, no obvious time differences were observed among the groups of rats finding the platforms in MWM experiment. However, after 96 h and 120 h SD, statistical analysis revealed a significant difference (*P* = 0.029) and a high significant difference (*P* = 0.007) between the M group and the N group, respectively, indicating that the ability of learning and memory of rats was markedly damaged after a long time of SD. Further, we applied GS to feed rats during SD. The latent period of GS-administrated SD rats was markedly lower than the M group and only slightly higher than that of the WP group, as well as higher than the normal group but with no statistical significance after 96 h and 120 h sleep intervention, indicating that GS could protect the damage of learning and memory of rats which was caused by long-term SD.

### 3.2. Biochemical Study

SD is an inherent stressor. The serum ACTH, TSH, and CORT levels of the model group were higher than those of the other groups (Table 2), suggesting that the hypothalamic-pituitary-adrenal (HPA) axis has been activated by SD. CA is associated with stress induced from psychological reactions or environmental stressors. The elevated serum CA in the model group reflected the pronounced sympathoadrenal activation induced by a well-established stressor method such as SD [28]. Comparison indicated that serum serotonin was markedly enhanced whereas serum testosterone was decreased in model group, which is consistent with the previous reports [29, 30]. The reduced serum testosterone level was associated with the inhibition of increased circulating serotonin and HPA axis activity, especially CORT concentration [31, 32]. GS intervention significantly reversed all biochemical parameters of SD rats, with no statistical differences from normal and WP groups. Thus, GS administration could effectively alleviate the symptom of SD.

### 3.3. Nitrogen Oxide (NO) Levels of the Brain

**Figure 1:**
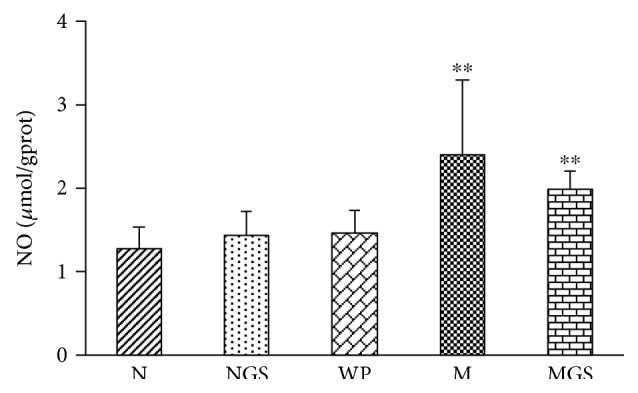
Brain nitrogen oxide (NO) levels of experimental animals. N: normal rats; NGS: normal rats fed by GS; WP: rats housed in wide platform; M: SD model rats; MGS: SD model rats fed by GS. Significant differences were determined by one-way ANOVA. ^∗∗^*P* < 0.01 versus the N group.

We also determined two important intermediates of free radical damage nitric oxide (NO) in the brain tissue (Figure 1). Compared with normal and WP groups, NO was significantly increased in model group, implying an elevated oxidative stress injury by SD. GS markedly decreased NO concentration. In fact, NO is a neurotransmitter and intracellular signaling molecule which was reported as a neuroprotective agent. Our study suggests that GS might prevent the free radical damage as well as protect the brain.

### 3.4. Serum Metabolomics of Sleep Deprivation and Total Ginsenoside Intervention

**Figure 2:**
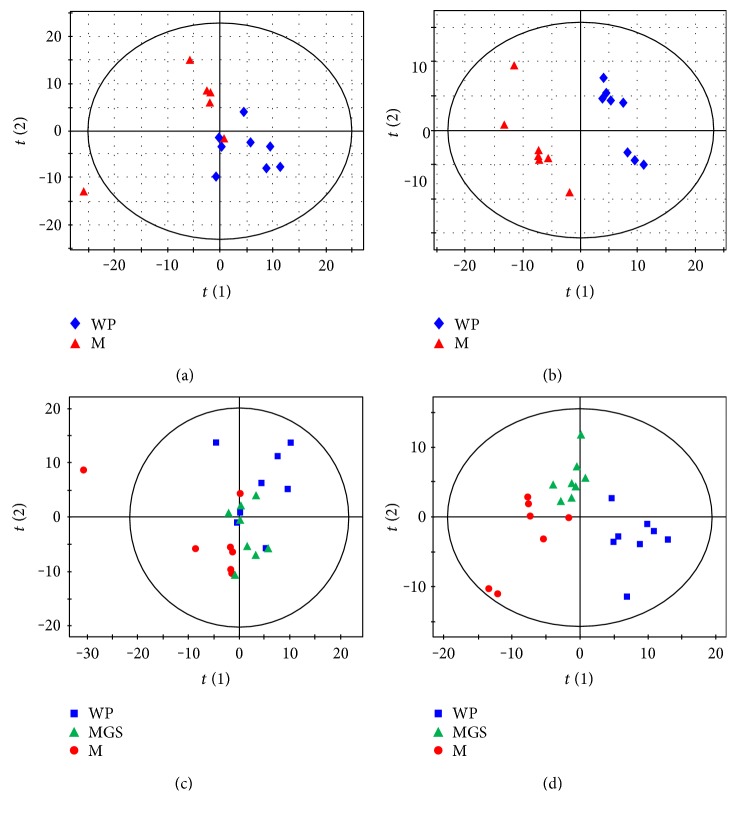
Score plots of multivariate statistical analysis on serum samples. (a) PCA score plot of wide platform (WP) group versus model (M) group, (b) PLS-DA score plot of wide platform (WP) group versus model (M) group, (c) PCA scores plot of WP, M, and MGS groups, (d) PLS-DA score plot of WP, M, and MGS groups.

No difference was found between the N group and the WP group by PCA, and also, no effective PLS-DA and OPLS-DA model could be established (figure not shown), indicating almost consistent serum metabolic phenotype between the N group and the WP group. Therefore, in comparison to brain metabolome, serum metabolome was insensitive to experimental environment variation. In order to keep identical in context, we selected WP group as the control group. A clear separation trend was found between model group and control group by PCA (Figure 2), suggesting more difference than that of the brain. PLS-DA score plot indicates a significant profile separation between control and model groups and that the administration of GS reversed the profile of model group towards control group. The results illustrated that the SD on rats could cause significant variations of serum metabolites and was alleviated by GS intervention.

Similar to the brain, 40 serum metabolites were statistically identified and structurally confirmed as differential metabolites responsible for SD (Table 3). These metabolites mainly are amino acids, hydroxyl acids, fatty acids, carbohydrates, and several indole-containing metabolites such as indoxyl sulfate, 3-indolepropionic acid, and serotonin. The intervention of GS caused the reverses of the levels of 23 serum metabolites, of which 15 metabolites showed statistical significance. All differential metabolites could be classified to 4 types. The type 1 and 2 metabolites are consisted of 23 metabolites of interest, potentially correlating closely to the intervention of GS.

### 3.5. Brain Metabolomics of Sleep Deprivation and Total Ginsenoside Intervention

**Figure 3:**
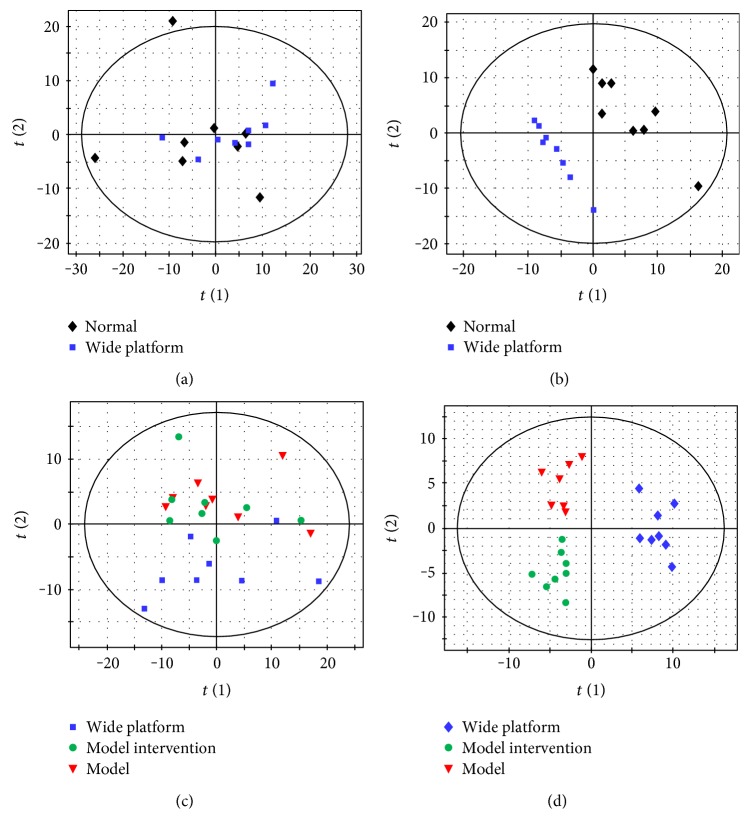
Score plots of multivariate statistical analysis on brain tissues. (a) PCA score plot of normal group versus wide platform (WP), (b) PLS-DA score plot of normal group versus wide platform (WP) group, (c) PCA score plot of WP, M, and model with GS intervention (MGS) groups, (d) PLS-DA score plot of WP, M, and MGS groups.

Metabolomic analysis based on GC/MS was performed to profile brain and serum metabolome of the rats following SD. No metabolic profile separation was observed between normal group and wide platform (WP) group by PCA score plot (Figure 3(a)), but a significant metabolic discrimination was revealed by PLS-DA score plot (Figure 3(b)) for brain tissue samples, indicating a considerable disturbance. We presumed that this distinction might be due to the experimental environment difference between normal housing and wide platform housing, and thus, WP group was ultimately selected as the control group when performing metabolomic study. There is a clear separation between WP group and model group in PC2 of PCA score plot (Figure 3(c)) and a further separation in PC1 of PLS-DA score plot (Figure 3(d)). Thus, our metabolomic study demonstrated a significant brain metabolic difference between model and control groups of SD.

Total ginsenosides (GS) are the extracts from *Panax ginseng*, a traditional herbal medicine which is used to improve physical and mental well-being and could effectively improve cognition and behavior on SD rats. Based on multivariate statistical analysis, it was found that MGS group could not be discriminated from model group by PCA (Figure 3(c)), but was well separated from model group by PLS-DA (Figure 3(d)), indicating that GS has significant effects on the brain metabolism of SD rats. Together with behavioral and biochemical results, this study showed that the brain injury caused by SD was alleviated by the administration of GS.

To identify the metabolites correlating with SD and GS administration, we performed a univariate statistical analysis such as FDR adjusted Student's *t*-test (*P* < 0.05) and a multivariate statistical analysis such as PLS-DA (VIP > 1) on whole brain metabolites. Totally, 39 brain metabolites were identified and structurally confirmed as differentially expressed metabolites responsible for SD (Table 4). SD principally resulted in the decrease of metabolites of glycolysis, pentose phosphate pathway (PPP), branched chain amino acids (BCAA, including valine, leucine, and isoleucine), tyrosine, and the amino acids related to potential antioxidation (cysteine, threonine, serine, methionine, and 4-hydroxyproline) and the increase of fat metabolism derivatives (glycerol 3-phosphate and 3-hydroxybutyric acid) and neurotransmitter (glutamic acid and aspartic acid) in the brain. Additionally, it was found that, compared to control group, adenosine and cytidine monophosphate were elevated but their products of degradation (uracil, inosine, hypoxanthine, and xanthine) were decreased in model group, suggesting a disordered nucleotide metabolism of SD. Overall, SD resulted in the disturbances of glucose metabolism (glycolysis, citric acid cycle, and PPP), fat metabolism, amino acid metabolism, and nucleotide metabolism.

Analyses on differential metabolites found that GS administration caused a reversion of a majority of brain metabolites (19 in total, 49%), of which 15 metabolites showed statistical significance. According to the concentration changes between groups, all differential metabolites could be classified to 6 types. The type 1 and 2 metabolites are consisted of 19 metabolites of interest. Analysis of correlation matrix showed high correlation of BCAAs and cysteine, PPP metabolites (except ribose 5-phosphate), and inosine and its metabolites (hypoxanthine and xanthine). Pathway analysis on all metabolites within groups also supported the importance of BCAA metabolism and PPP. The study indicated that GS principally acted on the disordered brain BCAA metabolism, PPP, and nucleotide metabolism of SD.

## 4. Discussion

**Figure 4:**
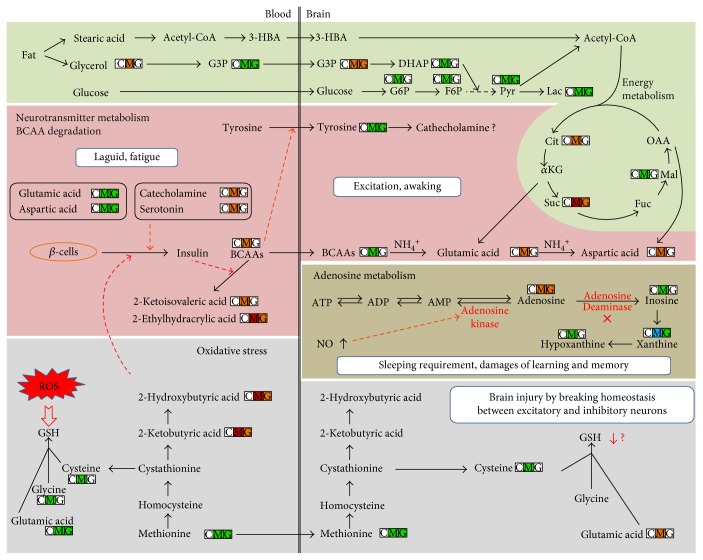
Proposed metabolic mechanism by SD and total ginsenoside intervention. The concentration changes are indicated in three small boxes with different colors and alphabets, where the box with “C,” “M,” and “G” represents WP group (as control group), model group, and GS intervention on model group. Red color indicates elevated level than WP group, while green color indicates decreased level than WP group. Red dash arrow represents suppression.

In this study, the animal model of SD was successfully constructed, behavioral and traditional biochemical parameters displayed that the ability of learning and memory of rats was markedly damaged after a long time of SD, and GS could protect the damage of learning and memory of rats, which was caused by long-term SD, and markedly reduce serum ACTH, TSH, and CORT levels and NO concentration of brain tissue. Our study suggests that GS administration could effectively alleviate the symptom of SD. Apart from the biochemical and behavioral effects of GS, metabolomics was applied to investigate changes of the comprehensive metabolic characters and biomarkers in serum and brain tissue on a rat model of SD. The metabolomic study implied that the pathways of energy metabolism, adenosine metabolism, branched chain amino acid (BCAA) and neurotransmitter metabolisms, and oxidative stress in serum and brain tissue were disturbed when SD happened. The intervention of GS could significantly change metabolic phenotype of model rats, where serum metabolome showed an obvious reverse of model group to control group. TG principally improved energy metabolism, BCAA metabolism, adenosine metabolism, and oxidative stress. In the metabolic networks (Figure 4), the metabolic changes associated with SD in the MGS group were analyzed.

### 4.1. Sleep Deprivation Caused Opposite Changes of Branched Chain Amino Acids and Neurotransmitters between the Blood and Brain

Branched chain amino acids (BCAAs, including valine, leucine, and isoleucine) are essential amino acids in mammal animals. Their catabolism usually contained two steps. The first step is reversible transamination catalyzed by the branched chain aminotransferase isozymes to produce glutamic acid (from *α*-ketoglutaric acid) and branched chain *α*-keto acids (BCKAs), such as *α*-ketoisovaleric acid. The *α*-amino group can be further transferred from glutamic acid to alanine and aspartic acid. The second catabolic step is oxidative decarboxylation of BCKAs, catalyzed by the branched chain *α*-keto acid dehydrogenase enzyme complex. In this study, SD resulted in the significantly elevated levels of valine and its degradation product *α*-ketoisovaleric acid, as well as markedly decreased *α*-ketoglutaric acid, glutamic acid, and aspartic acid in the blood, indicating a disordered valine catabolism probably at the second step in SD. Alternatively, the increased serum 2-ethylhydracrylic acid and isoleucine also indicated a dysfunction of isoleucine degradation with short/branched chain acyl-CoA dehydrogenase deficiency [33]. Our study suggested that SD possibly caused the disordered degradation and consequently the significant accumulation of BCAAs in the blood. The elevated serum serotonin might contribute to the increases of serum BCAAs after SD. The circulating serotonin is produced mainly in enterochromaffin cells of the gut and excreted into the blood. Recent study found that serotonin could control the release of both insulin and insulin-like growth factor and thus suppressed the release of insulin from the *β*-cells of the pancreas [34]. Previous study also indicated that plasma insulin level was decreased after 8 days of sleep restriction of male rats [35]. Additionally, we observed the significantly raised 2-hydroxybutyric acid and its precursor 2-ketobutyric acid in the blood following SD. Recent population study identified 2-hydroxybutyric acid as an early metabolic marker of type 2 diabetes by inhibiting the release of insulin from *β*-cells [36]. Insulin can stimulate the uptake and diminish BCAAs [37]. Therefore, in our study, the increased serum serotonin and 2-hydroxybutyric acid after SD could suppress the insulin release from *β*-cells and thereafter lowered the degradation of BCAAs.

The accumulation of plasma BCAAs by SD will lead to important metabolic consequence. Laboratory and epidemiologic studies suggested that SD might play a role in increased prevalence diabetes and/or obesity [38]. Recently, numerous studies proved that plasma BCAAs were highly positively correlated with insulin resistance and the biomarkers of early diagnosis and prognosis on type 2 diabetes and metabolic syndrome [39, 40]. Our investigation implied that the elevation of plasma BCAAs caused by SD possibly would contribute to the increased risk of diabetes and/or obesity. Generally, BCAAs are transported from the blood into the central nervous system (CNS) through a blood-brain barrier (BBB) by the large neutral amino acid transporter 1 (LAT1) for providing a major source of brain nitrogen [41]. LAT1 transports all of the large neutral amino acids, including BCAAs and aromatic amino acids (AAAs; tryptophan, tyrosine, and phenylalanine). LAT1 transporter was shared competitively by BCAAs and AAAs; thus, the excessive serum BCAAs (generally, their concentrations are significantly higher than AAAs in the blood) caused by SD will further suppress the transportation of circulating AAAs into the brain. Tyrosine is the precursor of CA (norepinephrine and dopamine), while tryptophan is the precursor of serotonin. It is well known that serum serotonin and CA could not pass through BBB system, so brain serotonin and CA could be synthesized by only utilizing AAAs in the brain. Such reductions in brain tyrosine concentration would reduce the synthesis and release in the brain. Although CA in the brain was not detected due to the limited intensity in this study, the significantly decreased brain tyrosine suggested the possible reduction in the brain. This deduction was also validated by another study [42]. Our study suggests that the elevated serum BCAAs by SD suppressed the transferring of AAAs from the blood to the brain so as to synthesize brain inhibitory neurotransmitters. As a consequence of SD, the increased inhibitory neurotransmitters (serotonin and CA) and decreased excitatory neurotransmitters (glutamic acid and aspartic acid) in the blood would cause body fatigue, whereas the lowered inhibitory neurotransmitters (serotonin and CA) and elevated excitatory neurotransmitters (glutamic acid and aspartic acid) in the brain would schematically maintain the waking of the brain. Interestingly, the levels of BCAAs in the brain were decreased after SD (Table 3). It is known that brain BCAAs transported from the blood are major nitrogen sources for production of two excitatory neurotransmitters glutamic acid and aspartic acid [43]. The excessive excitatory neurotransmitters would require a large amount of *α*-amino group from BCAAs, which ultimately reduced the levels of BCAAs in the brain. The significantly increased brain glutamic acid and aspartic acid might reflect the ongoing waking during SD. Our results indicate that SD caused the lower BCAAs and higher excitatory neurotransmitters (glutamic acid and aspartic acid) in the brain compared to control group, which are just opposite to those in the blood. In our study, abnormal level of BCAAs and neurotransmitters between the blood and brain in the model group was reversed by GS.

### 4.2. Dysfunction of Adenosine Metabolism

Adenosine is an important homeostatic sleep modulator and increasingly thought to play an important role in learning and memory. Sleep can efficiently conserve energy and increase ATP in brain. SD usually produces severe energy challenge and thereby causes increased breakdown of ATP and elevation of adenosine. Adenosine can also be converted back to AMP by adenosine kinase, an enzyme that converts adenosine to AMP, or irreversibly converted to inosine by adenosine deaminase, an enzyme degrading adenosine to inosine [44]. The extracellular adenosine level in the basal forebrain gradually increases during prolonged wakefulness or SD and acts to inhibit the basal forebrain wakefulness-active neurons [45–47]. Our study observed that SD significantly increased cerebral adenosine and attenuated its catabolic products (i.e., inosine, hypoxanthine, and xanthine), suggesting that the increased cerebral adenosine were associated with the inhibition of adenosine deaminase in SD. A recent genetic screening experiment proved that a genetic variant of the adenosine deaminase increased deep sleep in humans [48]. The adenosine-promoting sleep was correlated with an adenosine A1 receptor-mediated inhibition of glutamatergic inputs to cortically projecting cholinergic and GABA/parvalbumin neurons [49, 50]. Therefore, the increase of endogenous adenosine induced by SD might be mechanistically responsible for promoting sleep onset. Nitric oxide is a gaseous neurotransmitter and intracellular signaling molecule, which was reported as a neuroprotective agent [51]. In our study, SD resulted in markedly increased nitric oxide in the brain (Figure 1), possibly by the insistently increased excitatory input from the brainstem to the basal forebrain [52]. Recent studies proved that SD induced the production of inducible nitric oxide synthase-dependent nitric oxide in the basal forebrain and frontal cortex, further leading to the enhanced release of extracellular adenosine [52, 53] by inhibiting adenosine kinase [54] and/or decreasing ATP production via actions on the electron-transport chain and mitochondrial respiration [55]. However, we did not know if the inhibition of adenosine deaminase was correlated with nitric oxide. During SD, the site-specific accumulation of adenosine and NO in the basal forebrain resulted in an increased requirement of sleep [45, 56]. We proposed that the increased adenosine promotes the appetency of sleep and thus functions as a homeostatic sleep modulator. Adenosine has also other important influence on brain functions. One research demonstrated that astrocyte-derived adenosine and A1 receptor activity contribute to the sleep loss-induced deficits in hippocampal synaptic plasticity and hippocampus-dependent memory in mice [57]. Other studies also proved that the deficiency or selective inactivation of adenosine A_2A_ receptor significantly improved spatial recognition memory [58, 59]. Overall, adenosine metabolism disorder by SD not only enhanced the inherent requirement for sleep but also deteriorated learning and memory. In MGS group, the concentration of adenosine and nitric oxide returned close to normal, indicating that the therapeutic effects of GS may rely on the regulation of the dysfunction of adenosine metabolism.

### 4.3. Increased Energy Expenditure and Oxidative Stress in Sleep Deprivation

The important functions of sleep contain energy conservation and tissue restoration such as providing sufficient time to repair oxidative damage of cells and facilitate synthesis of some molecules for protecting against oxidative stress in rodents and humans [60]. Numerous studies have proved that SD resulted in increased energy expenditure [60–62]. We also observed that 96 h SD resulted in significant weight loss, accompanied with markedly increased food intake. Our metabolomic experiment demonstrated the significantly decreased concentrations of metabolites of glycolysis and pentose phosphate pathway in the brain, as well as the lower glucose (*P* = 0.092) and other related metabolites of glycolysis and TCA cycle in the blood in model group than control group (no statistical significance, data not shown). CORT is adrenal steroid hormones systemically released in response to stressors including SD. The elevated CORT secretion associated with 6 h SD promoted glycogenolysis and increased the circulation and brain glucose in response to energy demands [63]. Previous study indicated that 96 h SD resulted in the increase of hexokinase activity and the decrease of glucose-6-phosphatase activity in rat brain [64], suggesting the elevated activity of glucose metabolism. Another study of five nights of sleep restriction on rats also found a marked weight loss and decreased plasma glucose [65], suggesting excessive energy expenditure by SD. We proposed that the excessive energy demands by prolonged SD such as 96 hours caused the decreases of glycogen and glucose in the body and brain. The exhaustive glucose forced organisms to “shift” toward utilizing alternative available energy sources. Ketone bodies are the only endogenous circulating substrates that have been shown to contribute significantly to cerebral metabolism [66]. We observed the significantly increased serum glycerol, stearic acid, and 3-hydroxybutyric acid after SD, suggesting improved lipid metabolism. Recent study prompted that the low level of blood glucose possibly triggered the procedure of lipolysis and consequently decreased the body weight during SD [65]. Since long chain fatty acids could not be transferred across blood-brain barrier system, stearic acid was then directed to be oxidized and further forms ketone bodies in the liver, such as 3-hydroxybutyric acid, which can travel across the blood-brain barrier system and serve as a good energy source with several folds of increase in the brain during starvation [67]. However, we did not know if 3-hydroxybutyric acid could affect the recognition function. The increased cerebral 3-hydroxybutyric acid could be transformed to acetyl-CoA and infused to TCA cycle and thus supplies sufficient energy source during SD. Therefore, our study demonstrated that the cumulatively excessive energy demands during 96 h of SD caused the significant increase of fat utilization. SD also caused elevated oxidative stress, which raised the need for glutathione synthesis and depleted glutathione constituents. Previous study showed that 96 h paradoxical SD (also known as rapid eye movement sleep) caused significantly decreased level of glutathione in the hypothalamus but not in other brain regions [68]. Our work found that glutathione constituents like glycine, glutamic acid, and cysteine and their precursors such as methionine, serine, and threonine were uniformly decreased markedly both in the blood and in the brain (except glutamic acid as neurotransmitter in the brain) following SD. Although we could not detect homocysteine, an intermediate in the pathway of glutathione synthesis, from the blood and whole brain by untargeted metabolomics, other publications have proved that SD reduced the level of total plasma homocysteine [69]. Serum ophthalmic acid, an analogue of glutathione, is a sensitive indicator of hepatic glutathione depletion and proved as a new biomarker for oxidative stress [70]. 2-Aminobutyric acid is a key intermediate in the biosynthesis of ophthalmic acid. The elevated serum 2-aminobutyric acid in model group also suggested the increased oxidative stress by SD. Together, this work showed that SD resulted in elevated oxidative stress. The elevated oxidative stress, redressed by GS, produced substantial free radical damage. For instance, malondialdehyde (MDA), one of the most important intermediates of free radical damage, could suppress cerebral function, by breaking homeostasis between excitatory and inhibitory neurons, and was one of the major substances in the brain leading to fatigue [71]. In comparison to control group, the significantly increased cerebral MDA level indicated the brain injury in model group by oxidative attack, implying the onset of fatigue by SD. However, recent publication pointed that 3-hydroxybutyric acid substantially protected against oxidative stress by specifically inhibiting class I histone deacetylases and improving the expression of genes encoding oxidative stress resistance factors FOXO3A and MT2 [72]. The increased 3-hydroxybutyric acid during SD also helps in protecting against oxidative stress. In our study, the significantly decreased concentrations of metabolites of glycolysis, oxidative stress, and pentose phosphate pathway were redressed by GS in the MGS group compared with the model group (Table 4). This observation suggests that SD might perturb energy metabolism and oxidative stress and that the therapeutic effect of GS on SD may include the regulation of energy metabolism and oxidative stress.

## 5. Conclusions

In this study, a metabolomic method based on GC/MS has been developed to establish the metabolomic profiles of serum and brain tissue samples to investigate protective effects on SD rats of GS and its action mechanism. Pattern recognition with multivariate statistical analysis showed that a clear separation of model group and control group was acquired for serum and brain tissue samples; the MGS group showed a tendency of recovering when compared to the control group, which was consistent with behavioral and biochemical parameters. Totally, 39 and 40 differential metabolites were identified as potential SD biomarkers of brain tissues and serum samples, respectively. The properties of these biomarkers suggest that the mechanism of action of GS may involve in regulating the dysfunction of energy metabolism, BCAA metabolism, adenosine metabolism, and oxidative stress. Our work revealed that a metabolomic approach has been considered as a useful tool to reveal the pathogenesis of diseases and reveal action mechanism of TCM on the whole body.

## Figures and Tables

**Table 1:** Latent period of rats finding hidden platform in Morris water maze experiment (in seconds).

Group	D3 (training)	D4 (SD 24 h)	D5 (SD 48 h)	D6 (SD 72 h)	D7 (SD 96 h)	D8 (SD 120 h)
N	114.67 ± 9.39	80.76 ± 37.69	55.68 ± 31.41	32.05 ± 17.70	23.07 ± 13.12	13.80 ± 8.02
NGS	113.71 ± 11.01	83.26 ± 33.58	57.66 ± 22.88	33.40 ± 22.12	24.33 ± 19.39	14.07 ± 9.86
WP	115.18 ± 5.79	86.54 ± 19.75	57.73 ± 35.76	35.52 ± 15.90	29.78 ± 22.17	19.15 ± 11.78
M	112.66 ± 10.70	94.73 ± 29.35	70.53 ± 26.47	48.53 ± 25.96	34.40 ± 14.74^∗^	26.23 ± 15.21^∗∗^
MGS	111.23 ± 9.45	86.93 ± 24.68	57.82 ± 33.55	41.90 ± 19.80	28.41 ± 17.17	20.93 ± 11.57

From experimental day 3 to day 8 (the whole SD period), all rats received 2 times learning trials with 30 min interval in MWM. N: normal control rats; NGS: normal control rats with total ginsenoside (GS) intervention; WP: normal control rats housed on wide platform; M: SD model; MGS: SD model with GS intervention. Normal, WP, and model groups received normal saline solution; NGS and MGS received GS orally at a daily dose of 100 mg/kg of body weight from day 1 to day 8. Data were expressed as the latent time (seconds) for each rat finding the hidden platform in test trials, shown as mean ± SD. ^∗^*P* < 0.05 and ^∗∗^*P* < 0.01, compared to the N group (*n* = 8).

**Table 2:** Serum concentrations of ACTH, TSH, CORT, CA, testosterone, and serotonin.

Group	ACTH (pg/ml)	TSH (*μ*IU/ml)	CORT (*μ*g/ml)	CA (pg/ml)	Testosterone (pg/ml)	Serotonin (ng/ml)
N	154.28 ± 24.71	0.19 ± 0.03	22.18 ± 4.35	258.03 ± 39.97	420.53 ± 76.05	508.03 ± 69.72
NGS	156.44 ± 30.53^##^	0.18 ± 0.03^##^	25.27 ± 3.78^##^	258.94 ± 38.43^##^	427.69 ± 86.54^#^	515.19 ± 91.49^#^
WP	168.94 ± 34.98^##^	0.20 ± 0.03^#^	26.27 ± 6.42^#^	272.69 ± 41.88^##^	387.69 ± 46.36^#^	522.69 ± 62.73^#^
M	238.56 ± 50.39^∗∗^	0.27 ± 0.07^∗∗^	40.61 ± 13.87^∗∗^	327.69 ± 25.48^∗∗^	331.06 ± 46.34^∗^	606.06 ± 64.59^∗^
MGS	187.66 ± 41.68^#^	0.21 ± 0.04^#^	28.77 ± 6.06^∗^^, #^	296.44 ± 26.62^∗^^, #^	375.19 ± 35.03^#^	546.44 ± 42.47^#^

Significant differences were determined by one-way ANOVA. ^∗∗^*P* < 0.01 and ^∗^*P* < 0.05 versus the N group; ^##^*P* < 0.01 and ^#^*P* < 0.05 versus the M group. Data are mean ± SD, *n* = 8. NGS: normal animal administrated with total ginsenoside (GS) group; WP: wide platform group; M: SD model group; MGS: model administrated with GS group; ACTH: adrenocorticotropic hormone; TSH: thyroid-stimulating hormone; CORT: corticosterone; CA: catecholamine. Normal, WP, and model received normal saline solution; NGS and MGS received GS orally at a daily dose of 100 mg/kg of body weight from day 1 to day 8.

**Table 3:** Differential expressed serum metabolites.

Class	Serum metabolites	M/WP		GS/M		GS/WP	
		*P* value	log2	*P* value	log2	*P* value	log2
Type 1	Stearic acid	2.90*E*−02	0.33	1.48*E*−02	−0.35	8.52*E*−01	−0.02
	2-Ethylhydracrylic acid	2.09*E*−04	1.01	2.71*E*−02	−0.47	6.21*E*−04	0.54
	2-Ketobutyric acid	1.18*E*−04	1.90	4.82*E*−02	−0.61	6.77*E*−04	1.29
	2-Hydroxybutyric acid	1.68*E*−03	2.78	2.05*E*−02	−1.35	4.90*E*−03	1.43
	3-Hydroxybutyric acid	1.64*E*−04	1.22	1.25*E*−02	−0.48	3.63*E*−03	0.74
	2-Aminobutyric acid	8.61*E*−04	1.13	2.37*E*−02	−0.63	8.20*E*−03	0.50
	Thymine	9.82*E*−05	0.97	7.52*E*−03	−0.57	2.12*E*−02	0.40
	*α*-Ketoisovaleric acid	2.81*E*−02	0.52	4.60*E*−01	−0.22	2.69*E*−01	0.30
	Glutaric acid	8.24*E*−03	1.21	7.56*E*−02	−0.73	1.48*E*−01	0.49
	Leucine	4.94*E*−02	0.32	1.91*E*−01	−0.19	2.69*E*−01	0.13
	Valine	1.25*E*−02	0.43	8.77*E*−02	−0.27	1.13*E*−01	0.16
	Glycerol	1.03*E*−02	0.38	6.30*E*−02	−0.35	7.85*E*−01	0.04

Type 2	Alanine	4.63*E*−06	−0.65	1.76*E*−02	0.24	3.49*E*−06	−0.41
	Proline	6.29*E*−04	−0.72	2.03*E*−02	0.37	7.55*E*−03	−0.35
	4-Hydroxyproline	8.49*E*−06	−1.13	7.56*E*−03	0.43	7.45*E*−05	−0.70
	Serine	6.23*E*−04	−0.70	4.98*E*−02	0.26	3.45*E*−03	−0.44
	Cysteine	5.74*E*−04	−0.55	6.84*E*−03	0.46	5.21*E*−01	−0.09
	Glycine	3.78*E*−04	−0.50	1.08*E*−03	0.35	8.99*E*−02	−0.15
	Erythronic acid	1.19*E*−02	−0.27	1.27*E*−02	0.23	5.95*E*−01	−0.04
	3-Indolepropionate	3.00*E*−02	−0.60	6.26*E*−02	0.41	3.86*E*−01	−0.19
	2-Ketoglutarate	4.85*E*−02	−0.60	2.68*E*−01	0.17	1.14*E*−01	−0.43
	Indoxyl sulfate	2.30*E*−02	−0.46	3.55*E*−01	0.23	3.17*E*−01	−0.23

Type 3	Sedoheptulose	6.15*E*−03	−1.16	5.32*E*−01	0.13	5.68*E*−03	−1.03
	Fructose	2.52*E*−03	−0.88	9.45*E*−01	−0.01	1.28*E*−03	−0.89
	D-Gluconic acid	6.01*E*−03	−1.58	2.28*E*−01	0.20	5.38*E*−03	−1.39
	Glycerol 3-phosphate	1.68*E*−02	−1.21	1.09*E*−01	0.27	2.70*E*−02	−0.94
	Threonine	5.77*E*−07	−0.56	5.36*E*−01	0.05	2.87*E*−06	−0.51
	Methionine	9.37*E*−04	−0.36	1.40*E*−01	0.16	2.94*E*−03	−0.20
	Glutamic acid	3.41*E*−03	−0.94	2.61*E*−01	0.16	4.66*E*−03	−0.78
	Aspartic acid	7.49*E*−04	−1.12	2.41*E*−01	0.23	1.08*E*−03	−0.89
	Asparagine	3.30*E*−03	−0.80	1.91*E*−01	0.31	3.84*E*−03	−0.49
	Orotic acid	2.82*E*−03	−0.54	5.83*E*−01	0.08	4.15*E*−03	−0.46
	Ethanolamine	2.01*E*−03	−0.45	8.71*E*−01	0.02	1.07*E*−03	−0.43
	3,4-Dihydroxybutyrate	2.48*E*−02	−0.32	5.57*E*−01	0.04	4.65*E*−02	−0.27

Type 4	Serotonin	2.33*E*−02	0.46	6.16*E*−01	0.09	3.47*E*−03	0.55
	Cholesterol	9.41*E*−03	0.65	7.50*E*−01	−0.05	5.30*E*−04	0.60
	Inositol phosphate	7.17*E*−04	0.67	4.63*E*−01	−0.10	1.59*E*−05	0.57

**Table 4:** Differential expressed cerebral metabolites.

Class	Brain metabolites	M/WP		GS/M		GS/WP	
		*P* value	log2	*P* value	log2	*P* value	log2
Type 1	Citric acid	1.30*E*−03	0.15	1.60*E*−04	−0.23	1.32*E*−01	−0.08
	Glycerol 2-phosphate	3.01*E*−03	0.18	2.21*E*−02	−0.12	2.69*E*−01	0.05
	Cytidine monophosphate	1.26*E*−03	0.27	8.06*E*−03	−0.29	8.30*E*−01	−0.02
	Inositol	7.45*E*−07	0.28	2.11*E*−02	−0.11	2.63*E*−03	0.17

Type 2	Glucose 6-phosphate	6.86*E*−03	−1.13	6.64*E*−03	1.03	7.54*E*−01	−0.10
	Fructose 6-phosphate	9.04*E*−04	−0.99	4.18*E*−03	0.62	9.80*E*−02	−0.37
	Sedoheptulose 7-phosphate	6.06*E*−03	−0.89	5.09*E*−02	0.48	1.05*E*−01	−0.41
	Dihydroxyacetone phosphate	1.06*E*−02	−0.63	9.45*E*−02	0.33	1.37*E*−01	−0.29
	Ribose	1.83*E*−07	−0.50	1.06*E*−03	0.19	1.54*E*−04	−0.31
	Malic acid	1.31*E*−03	−0.13	1.25*E*−03	0.11	5.07*E*−01	−0.02
	Hypoxanthine	3.47*E*−03	−0.24	1.82*E*−02	0.12	1.02*E*−01	−0.12
	Xanthine	2.52*E*−03	−0.40	3.05*E*−02	0.14	2.56*E*−02	−0.27
	Uracil	2.65*E*−02	−0.14	4.24*E*−03	0.16	7.26*E*−01	0.02
	Inosine	2.89*E*−02	−0.20	2.44*E*−01	0.10	3.04*E*−01	−0.10
	Valine	3.77*E*−03	−0.16	4.37*E*−05	0.23	2.68*E*−02	0.07
	Leucine	2.82*E*−05	−0.20	1.43*E*−05	0.18	6.53*E*−01	−0.02
	Isoleucine	3.17*E*−05	−0.20	1.37*E*−04	0.18	6.34*E*−01	−0.02
	Cysteine	5.07*E*−03	−0.26	1.05*E*−03	0.27	8.60*E*−01	0.01
	Palmitoleic acid	2.63*E*−02	−0.27	3.25*E*−01	0.11	1.28*E*−01	−0.16

Type 3	Threonic acid	4.46*E*−03	−0.21	1.98*E*−02	−0.19	1.34*E*−06	−0.40
	4-Hydroxyproline	1.26*E*−05	−0.37	5.77*E*−03	−0.19	2.48*E*−08	−0.56

Type 4	Threonine	2.82*E*−09	−0.32	1.36*E*−01	−0.04	1.31*E*−11	−0.37
	Serine	8.57*E*−04	−0.13	4.13*E*−01	−0.03	2.34*E*−04	−0.16
	Methionine	2.65*E*−06	−0.37	6.25*E*−01	−0.02	1.28*E*−06	−0.39
	Fructose	1.75*E*−04	−0.26	7.91*E*−01	−0.01	1.63*E*−05	−0.28
	Pantothenic acid	3.87*E*−04	−0.23	6.90*E*−01	0.03	7.00*E*−03	−0.20
	Lactic acid	5.96*E*−03	−0.11	4.31*E*−01	−0.03	6.51*E*−04	−0.14
	Sedoheptulose	5.90*E*−07	−0.63	6.64*E*−02	0.13	3.57*E*−06	−0.49
	Ribulose 5-phosphate	6.18*E*−04	−0.70	2.30*E*−01	0.22	2.59*E*−03	−0.48
	Pyruvic acid	7.10*E*−05	−0.70	5.68*E*−01	0.08	3.45*E*−05	−0.62
	Tyrosine	8.13*E*−03	−0.48	9.04*E*−01	0.02	1.65*E*−02	−0.46

Type 5	Aspartic acid	3.65*E*−02	0.07	3.47*E*−01	−0.03	2.19*E*−01	0.04
	Glutamic acid	4.55*E*−02	0.10	6.97*E*−01	−0.02	6.84*E*−02	0.08
	Glycerol 3-phosphate	1.23*E*−05	0.60	3.31*E*−01	−0.07	1.98*E*−06	0.53
	Ribose 5-phosphate	2.35*E*−02	0.22	7.91*E*−01	0.03	3.16*E*−02	0.25
	Adenosine	4.42*E*−05	0.78	2.88*E*−01	0.22	8.52*E*−04	1.01

Type 6	Succinic acid	6.32*E*−06	0.96	9.93*E*−04	0.43	1.04*E*−09	1.39
	2-Aminobutyric acid	7.27*E*−03	0.29	5.91*E*−06	0.52	6.26*E*−11	0.81
	3-Hydroxybutyric acid	7.96*E*−06	0.30	1.39*E*−08	0.55	1.98*E*−11	0.85

## Abbreviations

SD: Sleep deprivation
GC/MS: Gas chromatography coupled to mass spectrometry
GS: Total ginsenosides
WP: Wide platform
MGS: Model + total ginsenoside group
MWM: Morris water maze
NO: Nitrogen oxide
ACTH: Adrenocorticotropic hormone
TSH: Thyroid-stimulating hormone
CA: Catecholamine
CORT: Corticosterone
PCA: Principal component analysis
PLS-DA: Partial least squares discriminate analysis
OPLS-DA: Orthogonal projections to latent structures discriminant analysis
UV: Unit variance
VIP: Variable influence on projection
BCAAs: Branched chain amino acids
PPP: Pentose phosphate pathway
BBB: Blood-brain barrier
AAAs: Aromatic amino acids
LAT1: Large neutral amino acid transporter 1.
